# The use of thymol, carvacrol and sorbic acid in microencapsules to control *Salmonella* Heidelberg, *S*. Minnesota and *S*. Typhimurium in broilers

**DOI:** 10.3389/fvets.2022.1046395

**Published:** 2023-01-04

**Authors:** Giovani Marco Stingelin, Ricardo Simões Scherer, André Costa Machado, Andrea Piva, Ester Grilli, Rafael Casarin Penha Filho

**Affiliations:** ^1^Farmabase Animal Health, Jaguariúna, Brazil; ^2^Vetagro S.p.A., Reggio Emilia, Italy; ^3^DIMEVET, University of Bologna, Bologna, Italy; ^4^Department of Veterinary Pathology, School of Veterinary and Agricultural Sciences, São Paulo State University, Jaboticabal, Brazil

**Keywords:** broiler chicken, intestinal health, antimicrobials, non-typhoidal *Salmonella* (NTS), essential oils, organic acids

## Abstract

**Introduction:**

The control of *Salmonella* spp. in poultry involves different biosecurity actions and lately has been complicated by the emergence of multidrug resistant serovars. The application of organic acids and essential oils has been used with different approaches due to the antibacterial properties as food preservatives. The use of these molecules in animal feed to control enteric pathogens is a major interest within the poultry industry.

**Methods:**

The use of a blend containing nature-identical compounds of sorbic acid (25%), thymol (9.5%) and carvacrol (2.5%) microencapsulated in a lipid matrix, was investigated in the present work, for the control of three *Salmonella* serovars (*S*. ser. Typhimurium, *S*. ser. Heidelberg and *S*. ser. Minnesota). Commercial broilers were challenged at 3 or at 33 days of age. Groups SH-1, SM-1 and ST-1, received treatment in the feed, at 2 kg/ton from 1–21 days of age and at 1 kg/ton from 35–42 days of age (last week), while groups SH-2, SM-2 and ST-2, were treated only during the last week receiving 2 kg/ton. Each treated group had an untreated control group, that was challenged at the same moment with the respective serovar (groups PCH, PCM and PCT). The challenge strains were enumerated in liver and cecal contents, weekly after challenge, at 7, 14, 21, 28, 35 and 42 days-of-age.

**Results and discussion:**

Significant reduction was noticed at 7 and 14 days of age in all groups that received treatment during the initial phase (*p* < 0.05). Moreover, the body weight was significantly higher at the last experimental day (*p* < 0.05) in chickens that received treatment at the initial and at the final growth stages.

## 1. Introduction

*Salmonella* spp. is a frequent etiological agent of foodborne infections. The presence of *Salmonella* spp. in the intestinal tract of apparently healthy poultry represents a high risk factor, considering that poultry may be an important carrier for the transmission to humans through the food chain, associated with consumption of contaminated meat, eggs or derived food (1). Food of animal origin, especially eggs and raw poultry meat, have been frequently associated to outbreaks of foodborne disease in humans caused by non-typhoid *Salmonella* (NTS) (2).

Although there are more than 2,600 different *Salmonella* serovars described, there is a tendency over time that few serovars are of higher epidemiological relevance. The Centers for Disease Control and Prevention (CDC) reported that only 5 serovars, including *S*. ser. Enteritidis and *S*. ser. Typhimurium, are responsible for more than 40% of all human salmonelosis cases (3, 4), during the last decade, in the United States. The emergence of novel serovars with higher epidemiological relevance in the poultry production systems has ocurred throughout this period, and many factors are involved in this relevance dynamics, such as virulence factors, environmental adaptability or resistance to antimicrobials.

*S*. ser. Minnesota has been frequently isolated from commercial poultry farms (10% to 30%) in Brazil, for the last 2 decades (5, 6). Other study shows that among *Salmonella enterica* serovars isolated from broilers in Brazil, *S*. ser. Minnesota was the most prevalent serovar and the horizontal transmission was identified as the main dissemination route (7). This same sorovar has gained high relevance in the last years due to environmental contamination of farms and also slaughterhouses (6). The isolation of *S*. ser Heidelberg is also a concern to the poultry industry. Although it is not frequently isolated from human cases of salmonellosis, this sorovar is reported as highly prevalent in poultry in Brazil and other countries, and has the capacity to contaminate and persist in slaughterhouses, carcasses and meat, increasing the risks of foodborne disease, often associated to multidrug resistant isolates (8).

The concerns with development of antimicrobial resistance, added to novel legislations banning or restricting antibiotic usage as growth promoters in food producing animals worldwide, raised the need for alternative approaches to control enteric pathogens, with alternative antimicrobial substances of natural origins, such as phytogenics, organic acids, enzymes, essential oils pre and probiotics (9, 10).

Essential oils are characterized as volatile or semi-volatile complex compounds extracted from plants (11). The different compounds may act synergistically or additively to exert their antibacterial properties. Disruption of bacterial cell wall and membrane is the most characterized antibacterial mechanism, leading to bacterial lysis, leakage and death (11).

Carvacrol and thymol are two promising molecules in essential oils that are present in oregano and thyme (11). These are potent antimicrobial agents acting in broad number of bacteria (12) and are generally considered safe for consumption (13). Both have been commonly used in cosmetic and pharmaceutical products, food preservatives and in animal feed.

The efficacy of organic acids in the prevention and treatment of foodborne pathogens have been evaluated in many studies (14, 15). Sorbic acid molecules in the non-dissociated form provides antimicrobial activity, which is influenced by pH (16). The optimal antimicrobial effect of sorbate increases if the pH is close to 4.75. Therefore, sorbate shows better antibacterial activity when used in environments with acidic pH values rather than higher pH ranges (17).

The limitations of organic acids and nature-identical compounds usage and efficacy against foodborne pathogens is related to their low resistance to stomach pH, limiting the dosage that reaches the intestinal tract where it is important to act. The methods for microencapsulation of organic acids and nature-identical compounds in lipid matrixes can improve the delivery of these compounds and overcome this problem (18). Thus, the present work, evaluated the microencapsulated organic acid and essential oils blend efficacy to control three *Salmonella* serovars in infected broiler chickens, including the emergent *S*. ser. Minnesota and *S*. ser. Heidelberg.

## 2. Material and methods

### 2.1. Bacterial strains

Three different *Salmonella* serotypes were used to challenge chickens according to the experimental design. The inocula were prepared separately with field isolates of *Salmonella* ser. Heidelberg (SH) or *Salmonella* ser. Minnesota (SM) or with *S*. ser. Typhimurium resistant to spectinomycin (Spec^r^) strain F98, kindly donated by Prof. Berchieri Jr. and maintained at Safe Animal Health Laboratory, Brazil. SH and SM were recently isolated from commercial broilers in Brazil and showed resistance to 3rd generation cephalosporins. Each strain was previously cultured on BGA plates for 24 h and one colony of each was separately streaked on separate tubes for culture of each serovar in Luria-Bertani (LB) broth (Oxoid, UK) grown for 24 h in a shaking incubator (100 rev/min) at 37°C. The cultures were centrifuged at 400 x*g* for 15 min and bacterial pellets were suspended in PBS Buffer to prepare each challenge inocula containing 2 *x*10^8^CFU/mL of the respective serovar, by measurement of the McFarland scale densitometer (Biomerieux, France), followed by plate counting to confirm the concentration.

### 2.2. Treatments

Treatments were based on commercial product containing organic acid and the nature-identical compounds of essential oils: sorbic acid (25%), thymol (9.5%) and carvacrol (2.5%), the compounds were microencapsulated in a lipid matrix of palm oil (63% hydrogenated fats), as previously described (18), (Avip^®^Protect; U.S. patent#7,258,880; EU patent #1-391-155B1; Vetagro SpA, Reggio Emilia, Italy). Treatments were performed according to the manufacturer instructions, as described in the experimental design, using 1 kg/ton (1 gr/kg) or 2 Kg/ton (2 gr/kg) directly mixed in the animal feed.

### 2.3. Experimental design and animal husbandry

A total of 210 chicks, from a commercial line of broilers were obtained at day-of-hatch from a hatchery in São Paulo State, Brazil and housed under controlled experimental conditions with water and food *ad libitum*. The chicks were randomly assigned to 9 groups with 30 chicks each, separated in different sanitary boxes with bedding (sterile wood shavings), at final concentrations of 12 per m^2^. Positive control groups, only received basal diet (feed without treatments) and broilers were respectively challenged with SH (group PCH), ST (group PCT) or SM (group PCM). Treated groups SH-1, ST-1 and SM-1 received feed with AviP^®^-Protect P, at 2 kg/ton, from 1 to 21 days-old and later, and at 1 kg/ton from 35–42 days-old. Groups SH-2, ST-2 and SM-2 only received feed with AviP-Protect P, at 2 kg/ton, from 35–42 days-old. The experimental procedures complied with the Ethical Principles in Animal Experimentation adopted by the Brazilian College of Animal Experimentation and were approved by the Ethical Committee on the Use of Animals.

### 2.4. Experimental challenge and sampling

At 3 days-of-age 20 chicks in treated groups SH-1, ST-1, SM-1 and 20 untreated chickens (control groups PCH, PCT and PCM) were challenged with 10^8^ CFU in 0.5ml of PBS buffer, cannulated directly into the crop, of the respective inocula, according to the experimental design. At 7, 14, 21, 28 days-of age, 5 chickens from each group were euthanized and samples of liver and caecal contents were harvested for *Salmonella* enumeration. At 33 days of age, 10 chickens from treated groups SH-1, ST-1, SM-1, SH-2, ST-2, SM-2 and remaining 10 birds of control groups PCH, PCT and PCM, were orally challenged by gavage with 10^8^ CFU/bird of the respective serovar and euthanized at 35 and 42 days-old to harvest liver and caecal content samples for *Salmonella* enumeration.

### 2.5. Bacterial counts in the tissue

Samples of caecal contents and pools of liver of challenged chickens were used to enumerate *Salmonella* (SH, ST or SM) per gram of organ (CFU/g) by plating serial dilutions of homogenized samples in Brilliant Green Agar (BGA, Oxoid, UK). In the absence of *Salmonella* growth, the vials containing the homogenized sample were enriched with Rappaport Vassiliadis (RV, Oxoid, UK) broth at 40°C for 24 h and plated. Values for bacterial numbers in positive enriched samples were considered 10^2^ CFU/g. Statistical analysis was performed with values transformed into log10 of CFU/g.

### 2.6. Evaluation of *Salmonella* shedding in the cloaca and litter

At 6, 13, 20, 27, 34 and 41 days-old, cloacal swabs were collected from 5 chickens per group and 1 g of poultry litter from each group were collected for inspection of *Salmonella* shedding. Samples were immediately placed in tubes containing RV broth and were plated on BGA, before and after enrichment, with incubation for 24 h at 37°C, to assess the presence of SH, ST or SM.

### 2.7. Evaluation of weight gain

The mean weight gain of broilers in each group was followed throughout the experiment, and before euthanasia for sampling, the body weight of 5 broilers in each group was evaluated, at 7, 14, 21, 28, 35 and 42 days-of-age.

### 2.8. Statistical analysis

Statistical analysis was performed with GraphPad Prism software version 8 for Windows. The individual values related to bacterial numbers (*Salmonella*) were compiled and the mean values for each group at the different moments evaluated were compared using two way ANOVA with comparative Fishers LSD test. The data of *Salmonella* isolation by cloacal swabs was evaluated by chi-square test, comparing treated against the respective control groups. Statistical significance was considered for *p* < 0.05.

## 3. Results

### 3.1. Evaluation of *Salmonella* counts in caecal contents and liver

**Figure 1:**
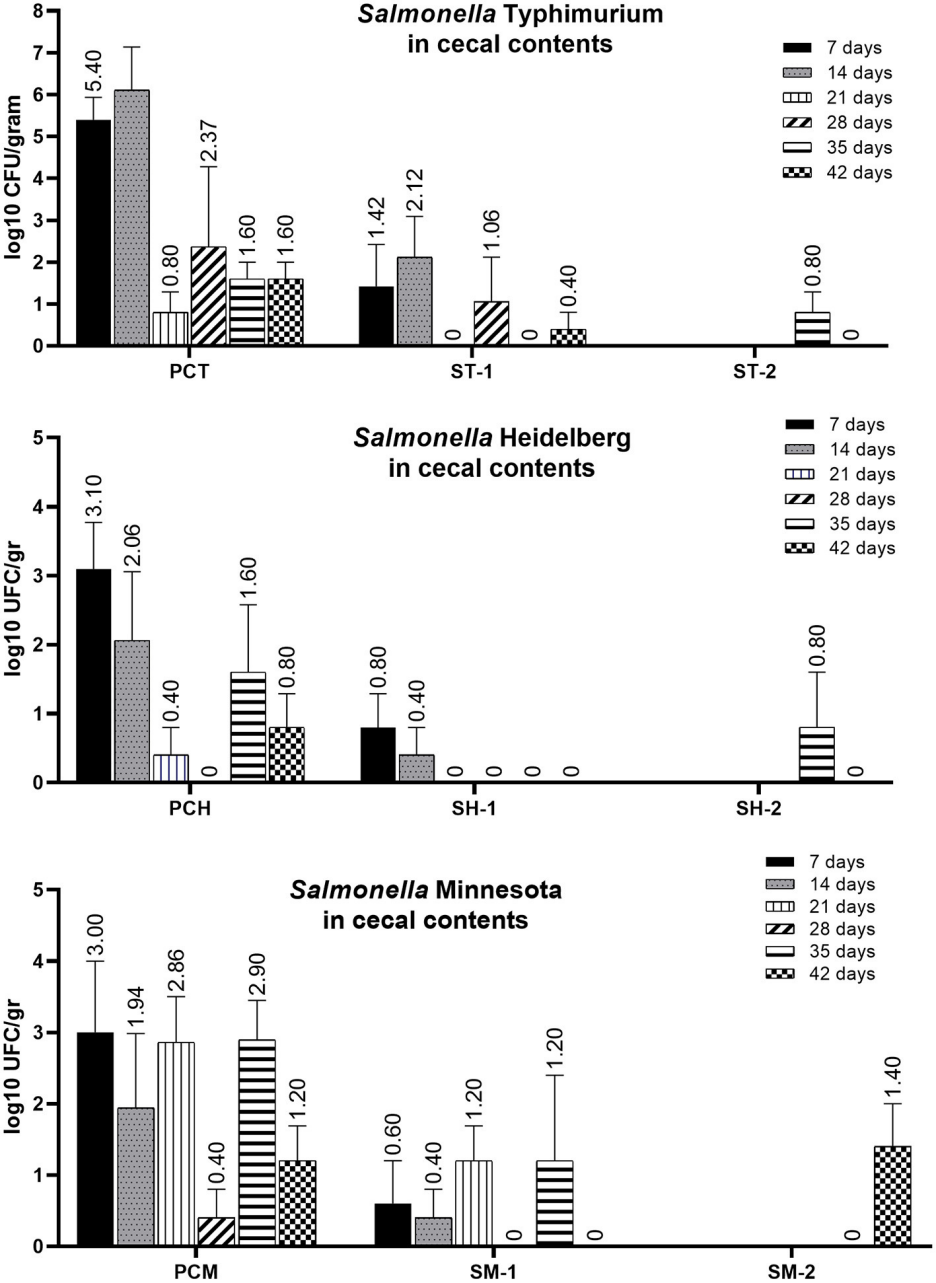
Bacterial numbers of SH, ST and SM in caecal contents, after challenge of 3 days-old and 33 days-old broilers in groups SH-1, ST-1 and SM-1 (treated from 1 to 21 days-of-age and from 35 to 42 days-of-age), SH-2, ST-2 and SM-2 (treated from 35 to 42 days-of-age) and positive control groups PCH, PCT and PCM (untreated).

**Figure 2:**
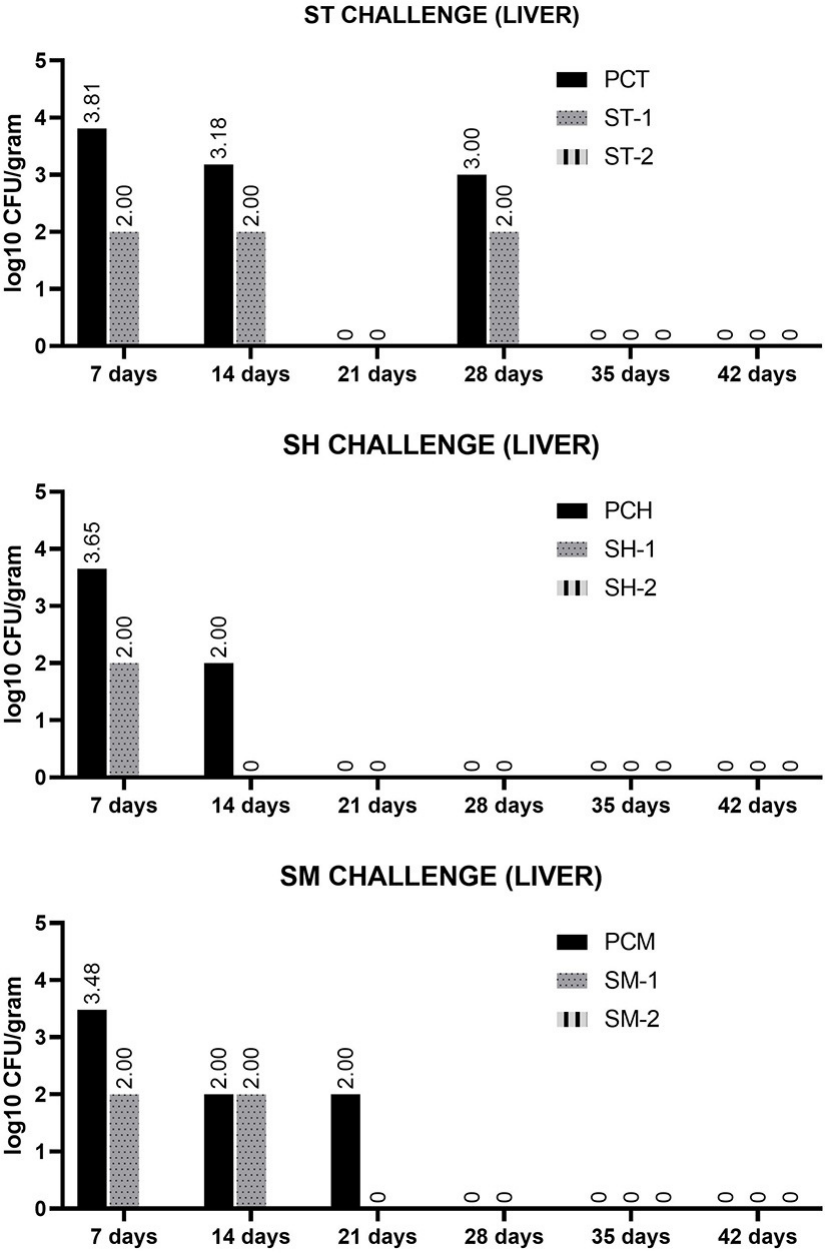
Bacterial numbers of SH, ST and SM in pools of 5 liver samples, after challenge of 3 days-old and 33 days-old broilers in groups SH-1, ST-1 and SM-1 (treated from 1 to 21 days-of-age and from 35 to 42 days-of-age), SH-2, ST-2 and SM-2 (treated from 35 to 42 days-of-age) and positive control groups PCH, PCT and PCM (untreated), at respective days-of-age. Considering this data were obtained from single pools for each moment, no statistics were performed for this data.

The results of SH, ST and SM counting in caecal contents are shown in Figure 1 and statistically evacuated in Table 1. The mean bacterial numbers in caecal contents in all control groups (PCH, PCT and PCM) was significantly higher at 7 and 14 days-of-age in comparison to all treated groups (SH-1, ST-1 and SM-1). The reduction of *Salmonella* in caecal contents at 35 and 42 days-of-age was also noticed in all treated groups, including SH-2, ST-2 and SM-2 in comparison to control groups, although was not significantly different at these moments (Table 1). The bacterial numbers in liver, are shown in Figure 2, all samples from treated groups were only positive after enrichment (considered as 2 log10), whilst in all positive control groups, the numbers were higher than 3 log10 in broilers at 7 days of age.

**Table 1:** Bacterial numbers of SH, ST and SM in cecal contents.

	**7 days**	**14 days**	**21 days**	**28 days**	**35 days**	**42 days**
SH-1	0.80^A^	0.40^A^	0^A^	0^A^	0^A^	0^A^
SH-2	-	-	-	-	0.80^A^	0^A^
PCH	3.10^B^	2.06^A^	0.40^A^	0^A^	1.60^A^	0.80^A^
ST-1	1.42^A^	2.12^A^	0^A^	1.06^A^	0^A^	0.40^A^
ST-2	-	-	-	-	0.80^A^	0^A^
PCT	5.40^B^	6.11^B^	0.80^A^	2.37^A^	1.60^A^	1.60^B^
SM-1	0.60^A^	0.40^A^	1.20^A^	0^A^	1.20^A^	0^A^
SM-2	-	-	-	-	0^B^	1.40^B^
PCM	3.00^B^	1.94^A^	2.86^B^	0.40^A^	2.90^A^	1.20^B^

Different letters indicate statistical difference in the same column comparisons were performed separately for each serovar (respective treated vs. control group).

### 3.2. Evaluation of *Salmonella* shedding by cloacal swabs

The monitoring of SH, ST and SM shedding in feces is shown in Table 2. A significant reduction of positive cloacal swabs was noticed in treated groups throughout the evaluation, especially at 6 and 13 days-of-age, when the bacterial burden was higher in positive control groups in comparison to treated groups. At 6 and 13 days-of-age, 5/5 and 3/5 broilers were positive for SM in group PCM, while in treated group SM-1, 1/5 and 2/5 were positive, respectively in the same moments. At 35 days-of-age, 3/5 were positive for SM in control group PCM, while no bird evaluated was positive in treated groups SM-1 and SM-2. The cloacal shedding of ST at 35 days of age was positive in 5/5 in control group PCT and in 3/5 and 4/5 in groups ST-1 and ST-2, respectively. The SH shedding at 35 days of age was positive in 3/5 in control group PCH, whilst in treated group SH-1 was 0/5 and group SH-2 was 1/5. At 42 days of cloacal shedding of ST, SH and SM were 0/5 in groups ST-1, SH-1 and SM-1, whilst, in control groups PCT, PCH and PCM, the positive clocal swabs were 2/5, 1/5 and 1/5, respectively for each serovar.

**Table 2:** Isolation of respective *Salmonella* serovars by cloacal swabs.

	**Total**	**14 days**	**21 days**	**28 days**	**35 days**	**42 days**	**Total (positives)**
SH-1	2/5	1/5	0/5	0/5	0/5	0/5	3/30^A^
SH-2	-	-	-	-	1/5	0/5	1/10^A^
PCH	1/5	2/5	0/5	0/5	3/5	1/5	7/30^A^
ST-1	1/5	1/5	0/5	2/5	3/5	0/5	7/30^A^
ST-2	-	-	-	-	4/5	1/5	5/10^B^
PCT	3/5	4/5	2/5	5/5	5/5	2/5	21/30^B^
SM-1	1/5	2/5	0/5	0/5	0/5	0/5	3/30^A^
SM-2	-	-	-	-	0/5	1/5	1/10^B^
PCM	5/5	3/5	0/5	0/5	3/5	1/5	12/30^B^

Groups that were evaluated only at 35 and 42 days, were compared with data from control groups from the same moments. Different letters indicate statistical difference at p < 0.05.

### 3.3. Evaluation of weight gain, clinical signs and mortality

**Figure 3:**
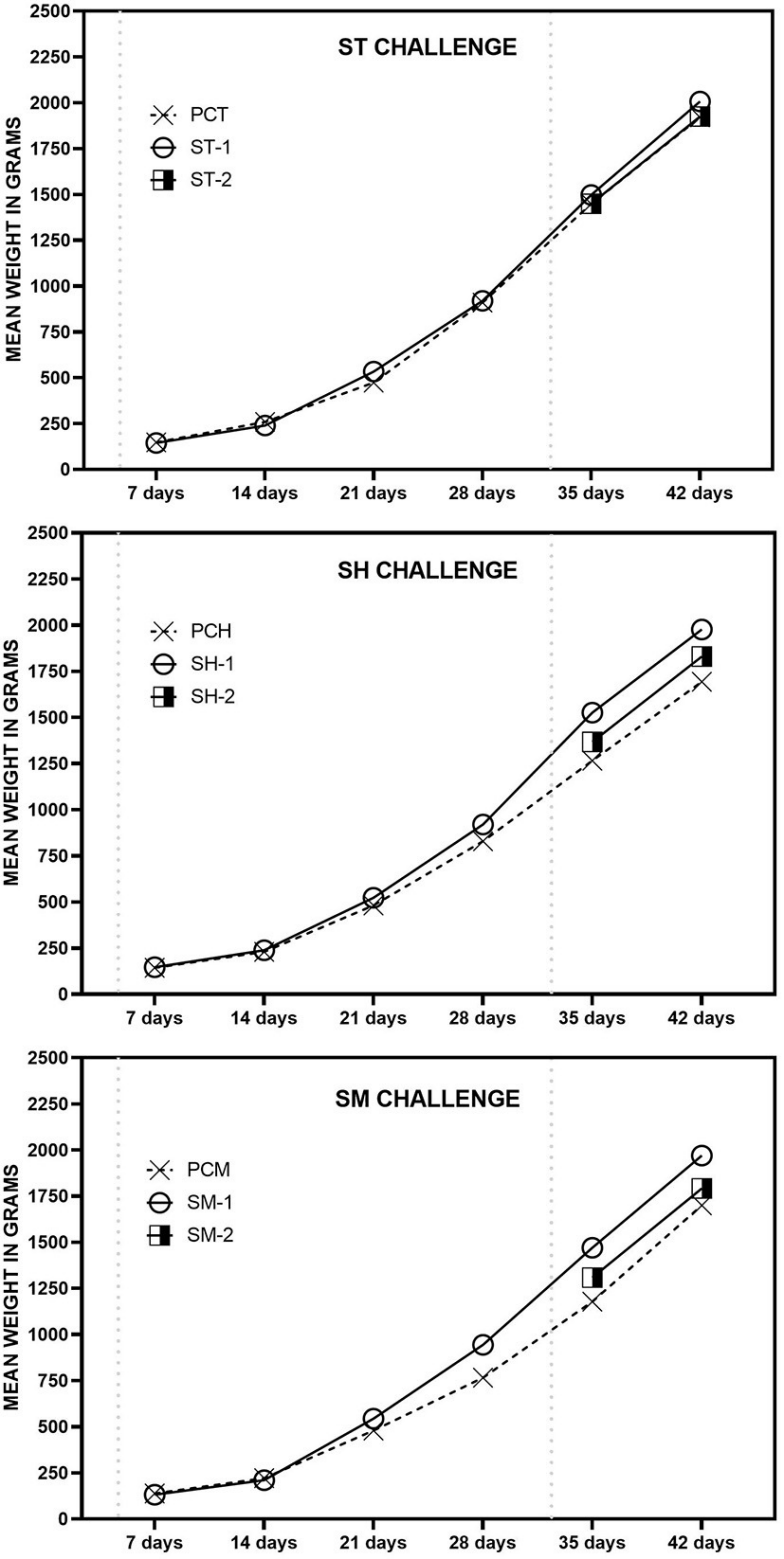
Mean body weight in grams of broilers challenged ST, SH or SM, in treated and untreated groups at respective days-of-age.

The mean body weight of broilers in each group is shown in Figure 3. It was noticed significant difference in the development of chickens, measured by body weight during the evaluated period. At 42 days-of age, the mean weight of broilers in groups that received treatment in the initial and final phase (Groups ST-1, SH-1 and SM-1), was significantly higher (*p* < 0,05) than the other groups, including the untreated positive control groups (PCT, PCH and PCM). No visible clinical signs of the disease were noticed, nor mortality was recorded in any of the groups after challenge.

## 4. Discussion

The use of organic acids as antimicrobials for food preservation and as animal feed additives has been evaluated for a long time. Initially, due to the antifungal properties of organic acid, these were used as food preservatives, to inhibit fungal growth and premature decomposition of food, however, in the last 10 years, they have also been studied as alternatives to antibiotic growth promoters. The improvement of feed conversion ratio and animal growth has been demonstrated before (19), as well as the capacity to reduce or prevent *Salmonella* colonization (20). Consequently, these molecules were since proposed as alternatives to the use of antibiotics as growth promoters in food-producing animals. Sorbic acid was first characterized by its antifungal activity, later, the antibacterial properties were also proposed, and ever since, it has been used in diverse industrial applications, such as food preservation, once it is more effective than benzoic or propionic acid, in higher pH conditions (21).

The use of sorbic acid has been characterized *in vitro* in previous study and the results demonstrated that at 0.025 to 0.1% and pH 5.2, it was capable to inhibit the growth of *Staphylococcus aureus* and *Salmonella* ser. Enteritidis in trypticase soy broth. Whereas, 0.08% was capable to inhibit the growth of yeasts, molds and coliforms in cheese a higher concentration of 0.3% was capable to delay the development of *Clostridium perfringens*, reducing gas formation and natural growth (21). In another study, the authors described that sorbic acid at 0.02% was the minimal inhibitory concentration for both *Salmonella* Hadar and *Salmonella* Enteritidis (18). The final concentration of sorbic acid in our study was 0.025 or 0.05% when it was used at 1 or 2 kg/ton of feed, respectively. Although the *in vivo* study may include other experimental variables than *in vitro* studies, our results showed a positive effect on this concentrations of sorbic acid in the feed, to reduce the serovars of *Salmonella* used for challenge (SH, ST and SM) in the intestinal tract of broilers. The isolates of SH and SM used in this study are field isolates and have the characteristics that are currently hampering the control of these bacteria and increasing their capacity to disseminate and persist in the poultry environment. Both isolates are resistant to 3rd generation cephalosporins and other antimicrobial classes, thus considered multidrug resistant isolates. Moreover, the presence of resistance genes is also associated to other virulence genes in the same resistance plasmids, that may increase the capacity of these bacterial to persist in all scales of the poultry industry, including breeders, farm environment, broilers and derived food. Despite these serovars show low relevance to human foodborne disease cases, compared to *S*. ser Enteritidis and *S*. ser Typhimurium, these are the most prevalent in the poultry industry and a major concern, as these are capable to contaminate the produced meat.

The bactericidal action of carvacrol was showed to be dose dependent and other authors verified that at 312 μg/mL it eliminated bacterial cells after 6 h and at 624 μg/mL the time reduced to 1 h. Carvacrol exhibited antibacterial and antibiofilm action against ST, and was suggested as a sanitizing agent for environments with food processing activities. The effects of carvacrol against ST was obtained with 500 μg/mL (0.05%), demonstrating bacterial eradication after 30 min of exposure at 22°C (22).

Human infections with *Salmonella* are mainly associated with consumption of contaminated food, of which, poultry and their derived products are frequently pointed as the main source, resulting in isolated cases or outbreaks of variable degree of severity, depending on the pathogenicity and invasiveness of the serovar (23). Different essential oils show synergic action in their antibacterial effects, consequently the efficacy of essential oil blends has shown to be higher than single essential oil preparations (24). The bacteriostatic effect of organic acids is dependent on various factors, such as source of origin and concentration of the preparation (25). The feeding method *in vivo*, is also considered and important factor that can interfere in the efficacy (26). Considering these synergistic effects, the novel preparations for use in environments or in animal feed (*in vivo*), demonstrate improved efficacy as antimicrobials, when the combination of essential oils and organic acids is used (27).

Essential oils have mechanisms of action that are not fully elucidated, however the lysis of the bacterial cell wall was demonstrated as the main mechanism of thymol to kill ST (28). Scanning electron microscopy characterized this mechanism of action demonstrating that both carvacrol or thymol were able to disrupt ST cell wall, with a complete loss of membrane integrity (29). Both carvacrol and thymol have hydrophobic nature, thus these compounds are able to integrate the bacterial cell wall, leading to disturbance in the functions and consequently disruption of this part (30).

The inhibition of efflux pumps, may increase bacterial susceptibility to antimicrobials, including organic acids and essential oils, and this mechanism is also proposed as another antibactial route of action, together with unbalanced ATP metabolism altering cell function, interference in protein synthesis, disturbances in cytoplasmic pH and quorum sensing (31).

Efflux pumps are present in both Gram-negative and Gram-positive bacteria, and are associated to excretion of toxic metabolites, including those produced by the bacteria or external substances such as antimicrobials, decreasing toxic intracellular levels and allowing bacterial survival and growth (32). Efflux pumps structures are able to remove antimicrobials from the cytoplasm due to their poly-substrate specificity, at some degrees, when the removal is accelerated and efflux pumps are overexpressed, the resistance to certain antimicrobials may happen by this mechanism (33). Otherwise, inhibition of efflux pumps may be a mechanism to increase susceptibility and this is currently being investigated as an alternative method to potentiate treatments and overcome antimicrobial resistance (34).

Carvacrol and thymol may additionally cause inhibition of efflux pump and consequent disturbances in bacterial metabolism. Previous study showed that thymol has a stronger inhibitory activity than carvacrol against different isolates of ST, using MIC values that ranged from 32 to 64 μg/mL. *Salmonella* Minnesota has been isolated in different sources of samples investigated, including animal-producing farms, food and plants (35). Moreover, the current isolates demonstrate increasing levels of resistance to important classes of antimicrobials, such as extended-spectrum cephalosporins (8). Considering that this resistance mechanism is highly effective through the production of antimicrobial inactivating enzymes, specific for the substrate, the search for novel alternatives for pathogenic bacteria control, that do not select or contribute to the maintenance of the current high levels of resistance genes in field isolates is very important. Although more data is still necessary to characterize the action of the organic compounds *in vivo* and its efficacy to kill bacteria with different acquired resistance mechanisms as occurs in the fields.

Recently, (36) demonstrated that the use of carvacrol together with thymol in male broilers feed during the initial and final experimental periods, improved feed consumption and conversion ratio and body weight gain. The authors concluded that the improvement of performance was related to the synergy between thymol and carvacrol, which was also directly related to increased feed intake (36). The development of nature-identical compounds contributed to improve the quality of these substances, however, the microencapsulation was a crucial step in the development of effective treatments to overcome the intestinal tract barriers. The microencapsulation of the active principles enabled this treatment to be released at the right portion of the intestinal tract, protecting the blend from the action of gastric juice. Furthermore, the microcapsules are able to control the release the active compounds (37). In the present study, no clinical signs or mortality was noticed in any of the groups after challenge, although the use of these molecules positively interfered in the final body weight of the chickens in comparison to the untreated groups. Despite the low interference of the challenge in the behavior of the chickens, feed intake may continue the same after challenge with non-typhoidal *Salmonella*, however, constant treatment and reduction of *Salmonella* loads in the intestinal mucosae may reduce intestinal damage and consequently improve feed conversion ratio in comparison to untreated chickens, which develop the natural course of intestinal infection and inflammation.

Considering that *Salmonella* main proliferation and persistence site is the ceca, it is important that the treatments can reach the desired concentrations at this location. The cecal contents may keep *Salmonella* for long periods and depending on the serovar, these bacteria may be found after 28 days at this site. The microencapsulated compounds used in the present treatment showed a relevant action in the control of *Salmonella* in the cecal contents and in the shedding of the bacteria, evaluated by cloacal swabs. The treatments significantly reduced the presence of the three serovars evaluated in the cecal contents of broilers, consequently a lower clocal shedding was also noticed. As noticed, the recovered bacterial loads of each sorovar used in the challenge, did not show the same dynamics of infection among serovars, and these results may shed light to the understanding of how different serovars disseminate or persist in poultry flocks. The analysis of positive control groups, that had no interference of treatments in the infection course, showed that ST has higher capacity to replicate in the ceca during the acute infection period, considered to occur up to 14 days after challenge, reaching mean values of 6,11 CFU/gr. The bacterial loads of other serovars reached maximum levels of 3.1 and 3 CFU/g of *Salmonella* Heidelberg and *S*. Minnesota, respectively, in the acute period. These observations may also be associated to the efficacy of the treatment to different serovars. As noticed, in group SH-1, treatment significantly reduced the *S*. Heidelberg numbers, and this serovar was not isolated after 14 days, in this group. Otherwise, ST and SM, showed a more persistent behavior, and were still isolated in reduced numbers at 21 or 28 days of age. Even though ST and SH are classified in the same serogroup B, it was noticed that both can demonstrate different infection behaviors. These differences are expected as each serovar has different genetic background, and not only the presence but the expression levels of virulence genes, may differ among them, consequently resulting in different levels of colonization, invasiveness, replication and even resistance to bacterial clearance by macrophages (38). These factors may be capable to modulate the intestinal and systemic infection of each serovar, thus resulting in different bacterial numbers found in feces and internal organs. As noticed in liver and cecal contents samples, evaluated in the present work. The other factor that may have contributed for reduced infection loads in the cecal contents was the body weight, once, as noticed, after the infection of chickens at 33 days of age, the bacterial loads recovered were lower than recovered from chicken challenged at 3 days of age. However, it did not positively interfere in the positivity rates of cloacal swabs at this final period, as shown in Table 2.

Moreover, the use of this treatment in the feed significantly sustained the growth of the birds after a high challenge dose. Considering the results obtained in this study we noticed the positive effects on the use of microencapsulated Thymol, Carvacrol and Sorbic acid in the control of emergent serovars of *Salmonella* in broilers. Moreover, the effects on *Salmonella* shedding may contribute to the reduce the contamination in the poultry environment and in carcasses in the slaughterhouses. Thus, this treatment demonstrated a good capacity to protect the intestinal health of commercial broilers.

## Data availability statement

The raw data supporting the conclusions of this article will be made available by the authors, without undue reservation.

## Ethics statement

The animal study was reviewed and approved by Ethics Committee in the Use of Animals, UNESP, Jaboticabal.

## Author contributions

GS has contributed from the elaboration to the development, analysis of the study results, and review of the manuscript. RS and AM have both contributed to the execution of methods and manuscript review. AP and EG have both contributed to the study proposal, financing, and manuscript review. RP has contributed to the financing, analysis of results, and writing of the manuscript. All authors contributed to the article and approved the submitted version.
